# Supplementary material for: Observation of multi-order polar radial vortices and their topological transition
Source: Nat Commun. 2025 Mar 21;16:2804. doi: 10.1038/s41467-025-58008-w (PMC11928551; doi:10.1038/s41467-025-58008-w)
Supplement: Supplementary file 1 — Supplementary Information [file 41467_2025_58008_MOESM1_ESM.pdf]

Supplementary Materials for

Observation of multi-order polar radial vortices and their topological transition

Wan-Rong Geng[1,†], Xiangwei Guo[2,†], Yin-Lian Zhu[1,3], Desheng Ma[4], Yun-Long Tang[5], Yu-Jia Wang[5], Yongjun Wu[2], Zijian Hong[2,*], Xiu-Liang Ma[1,6,7,*]

[1]Bay Area Center for Electron Microscopy, Songshan Lake Materials Laboratory; Dongguan, 523808, China.

[2]State Key Laboratory of Silicon and Advanced Semiconductor Materials, School of Materials Science and Engineering, Zhejiang University; Hangzhou, 310058, China.

[3]Hunan University of Science and Technology; Xiangtan, 411201, China.

[4]School of Applied and Engineering Physics, Cornell University, Ithaca, NY 14850, USA.

[5]Shenyang National Laboratory for Materials Science, Institute of Metal Research, Chinese Academy of Sciences; Shenyang, 110016, China.

[6]Institute of Physics, Chinese Academy of Sciences; Beijing, 100190, China.

[7]Quantum Science Center of Guangdong-HongKong-Macau Greater Bay Area (Guangdong); Shenzhen, 510290, China.

†Authors contributed equally to this work.

*Correspondence should be addressed to Z. H. (Email: hongzijian100@zju.edu.cn) and X. L. Ma (Email: xlma@iphy.ac.cn).

Table-of-Contents

Supplementary Table 1
Supplementary Fig. 1-Fig. 18

Supplementary Table 1| The sizes of the nanostructures for different polar topological states.

Types	One-order radial vortex	One-order radial vortex	Two-order radial vortex	Three-order radial vortex
**Diameter (nm)**	100	200	350	400
**Island height (nm)**	3	4.5	14.5	14.5
**SMR height (nm)**	-	2.5	11.5	11.5

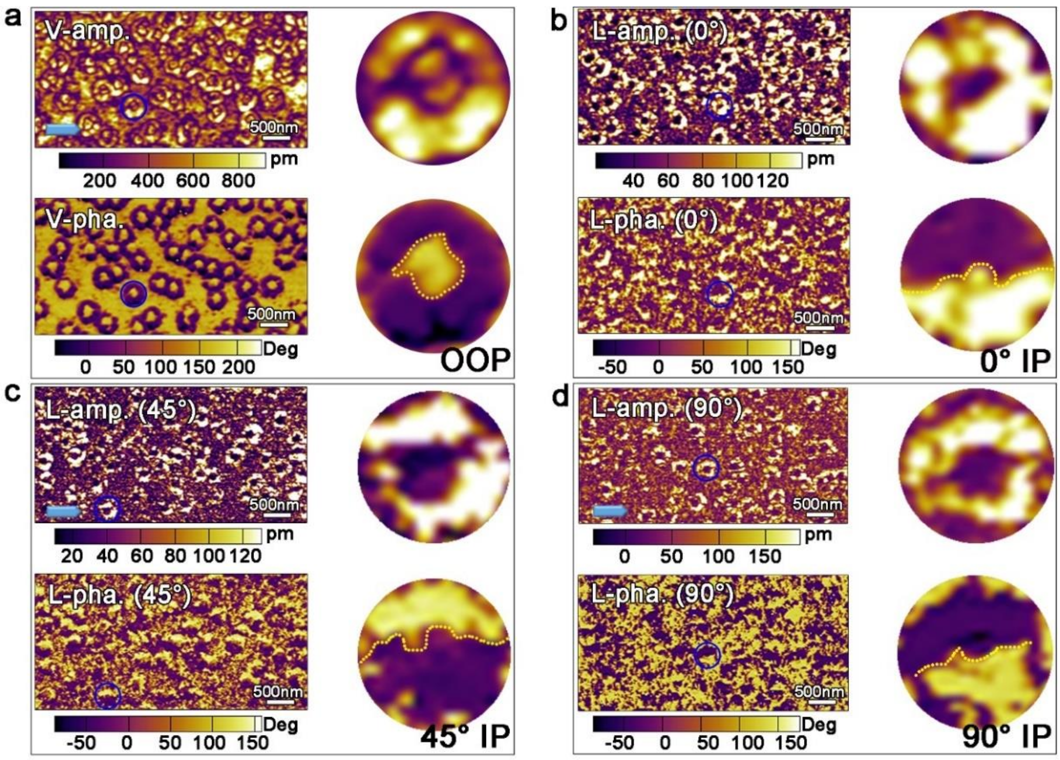

Supplementary Fig. 1| Detailed PFM analyses of BFO nanoislands. a, The V-amp. (top panel) and V-pha. (bottom panel) images of the BFO film. **b-d**, The L-amp. (top panel) and L-pha. (bottom panel) images of the BFO film with the sample rotation for 0° (b), 45° (c) and 90° (d), respectively.

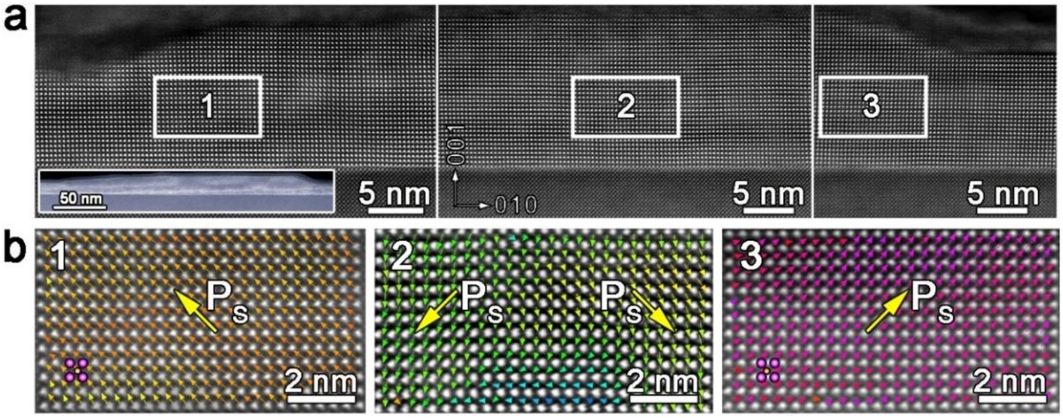

Supplementary Fig. 2| Detailed cross-sectional TEM analyses of the polarization distribution in nanoislands. **a**, A cross-sectional atomic-resolved HAADF-STEM image viewed along the [100] direction of BFO. Inset is the low-magnification HAADF-STEM image for one nanoisland. **b**, Polarization distribution of BFO corresponding to the rectangles numbered 1, 2 and 3 in (a), respectively.

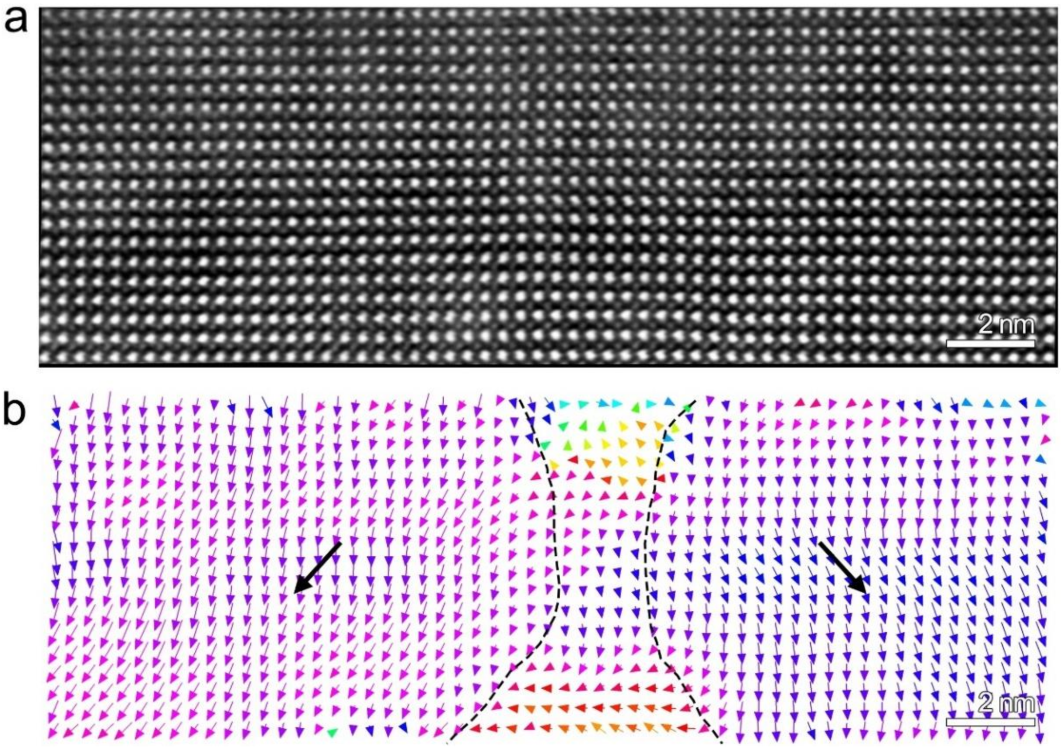

Supplementary Fig. 3| Polarization distribution at nanoisland cores. a, Cross-sectional atomic-resolved HAADF-STEM image. **b**, Corresponding polarization vector map, displaying the polarization distribution at the core of nanoisland.

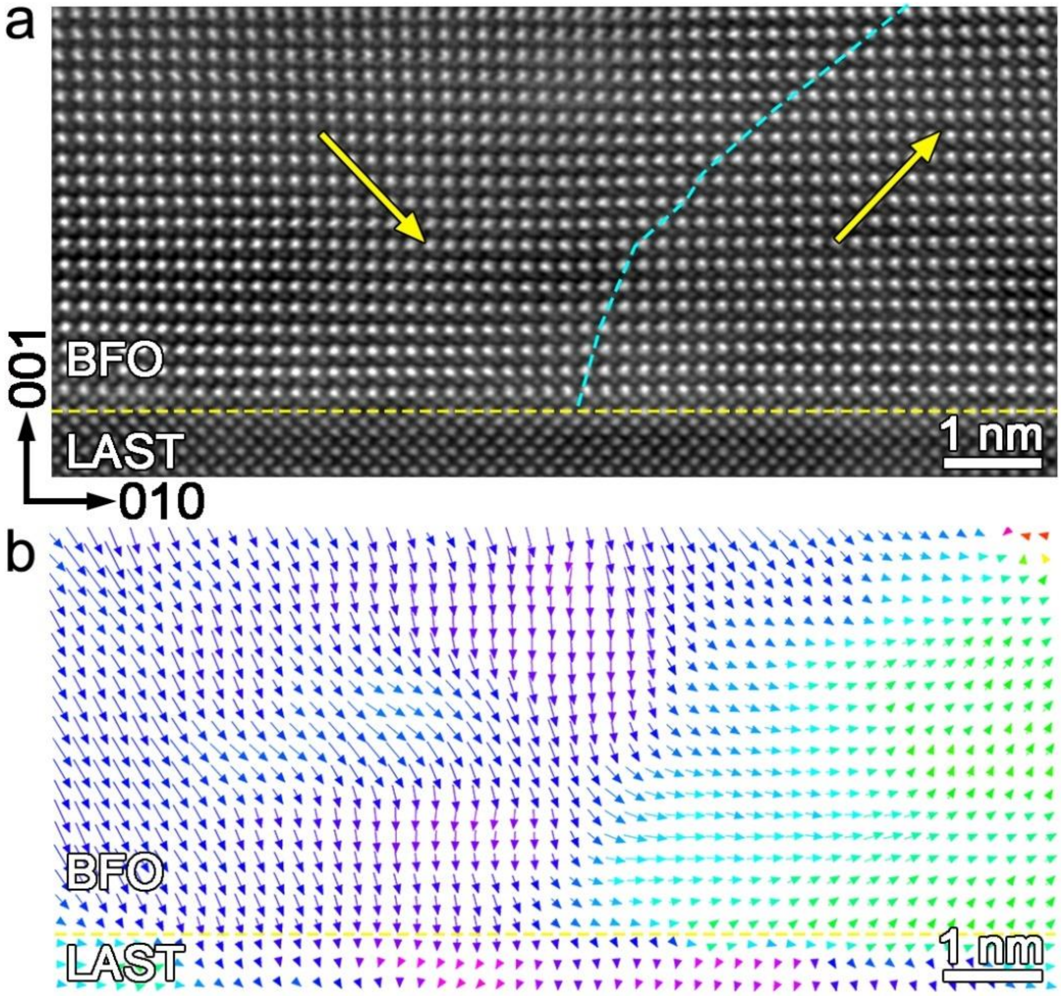

Supplementary Fig. 4| Polarization transition from nanoisland core to nanoisland edge. **a**, Cross-sectional atomic-resolved HAADF-STEM image. **b**, Corresponding polarization vector map, displaying the polarization transition from downward direction to upward direction.

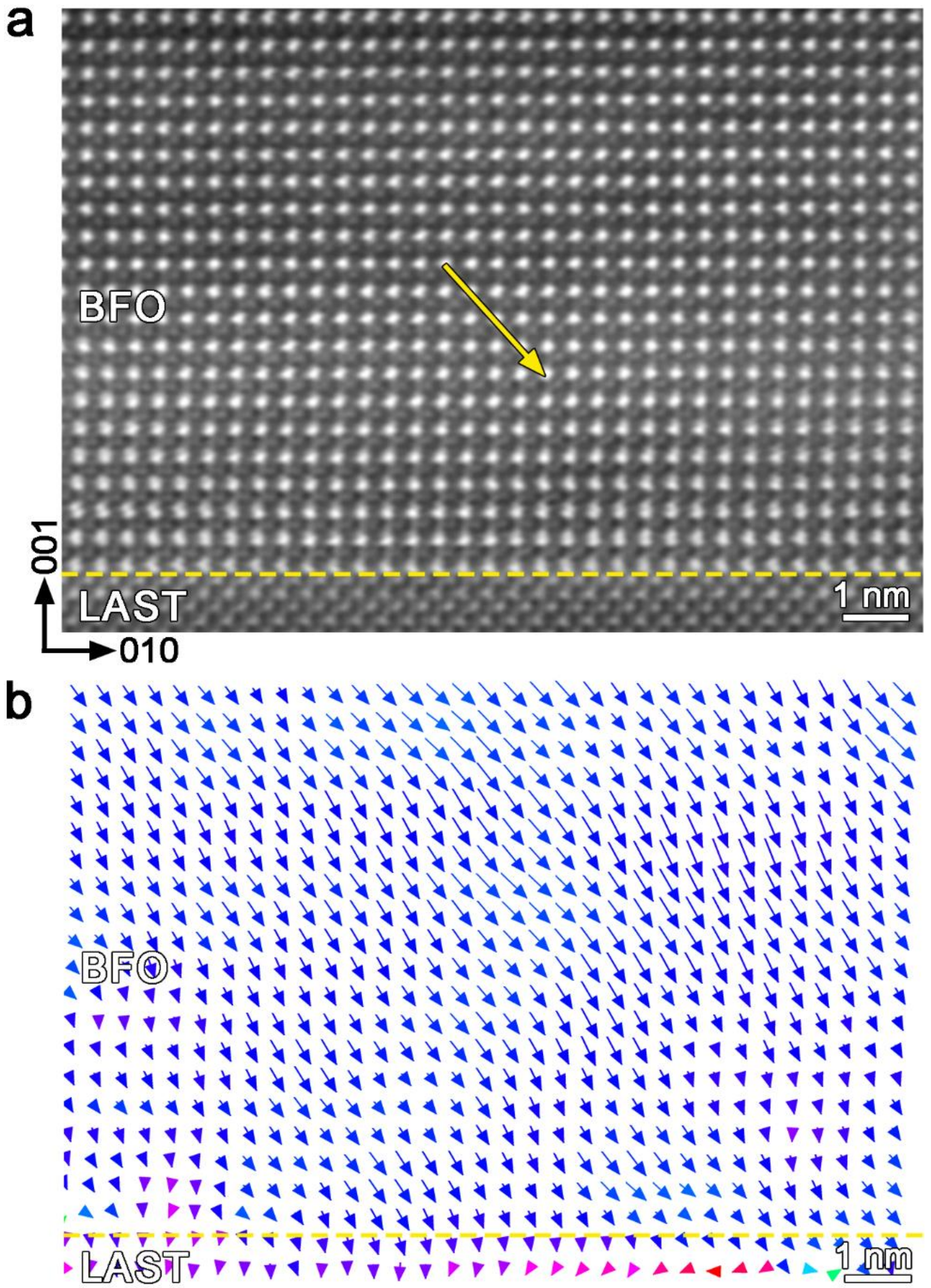

Supplementary Fig. 5| Polarization distribution of surrounding matrix region. a, Cross-sectional atomic-resolved HAADF-STEM image. **b**, Corresponding polarization vector map, displaying the downward out-of-plane polarization.

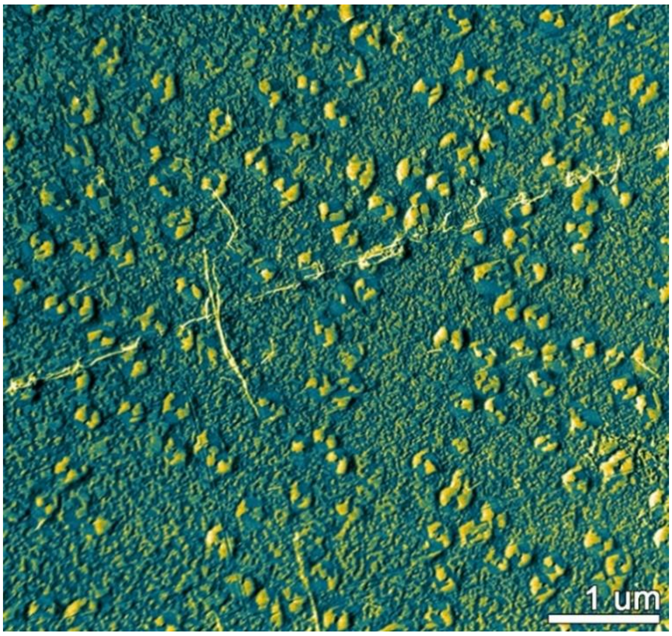

Supplementary Fig. 6| HAADF-STEM image of the BFO film, displaying the four-quadrant polarization distribution in nanoislands.

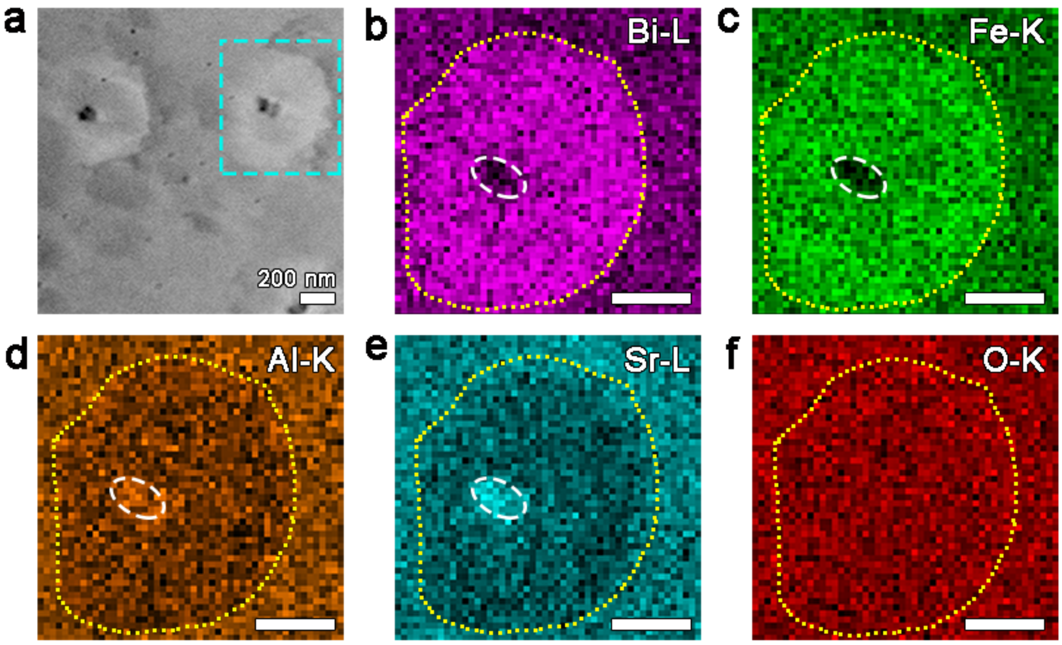

Supplementary Fig. 7| Elemental distribution in one nanoisland. a, Low-magnification HAADF-STEM image, displaying the nanoislands in the BFO film. **b-f**, EDS elemental maps of Bi-L (b), Fe-K (c), Al-K (d), Sr-L (e) and O-K (f) corresponding to the cyan rectangle in (a).

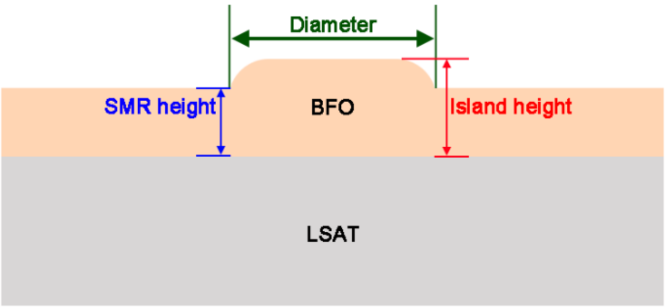

Supplementary Fig. 8| Schematic showing the size definitions of one nanoisland.

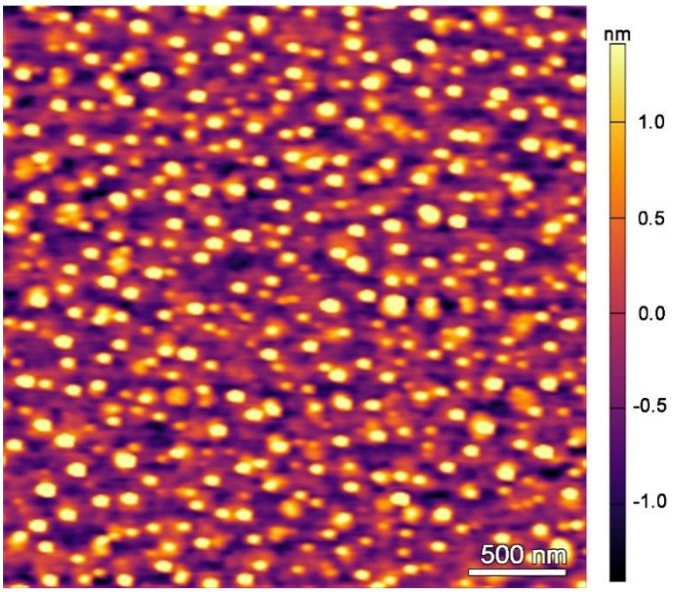

Supplementary Fig. 9| Topography image corresponding to the region in Fig. 3a-b.

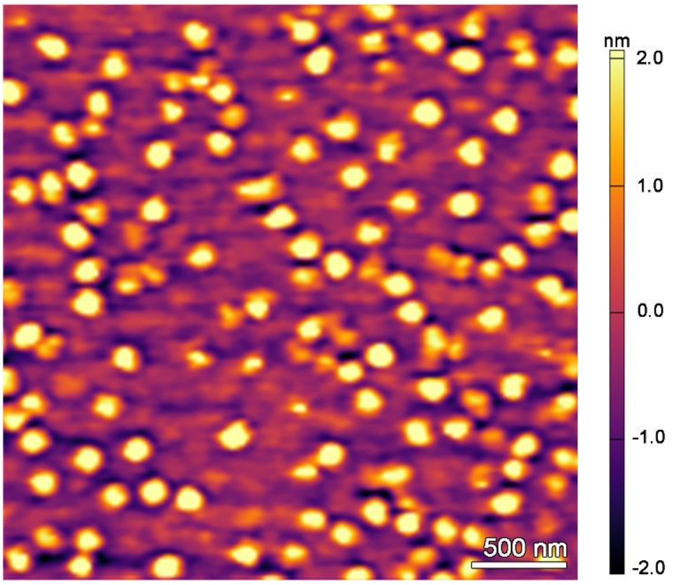

Supplementary Fig. 10| Topography image corresponding to the region in Fig. 3e-f.

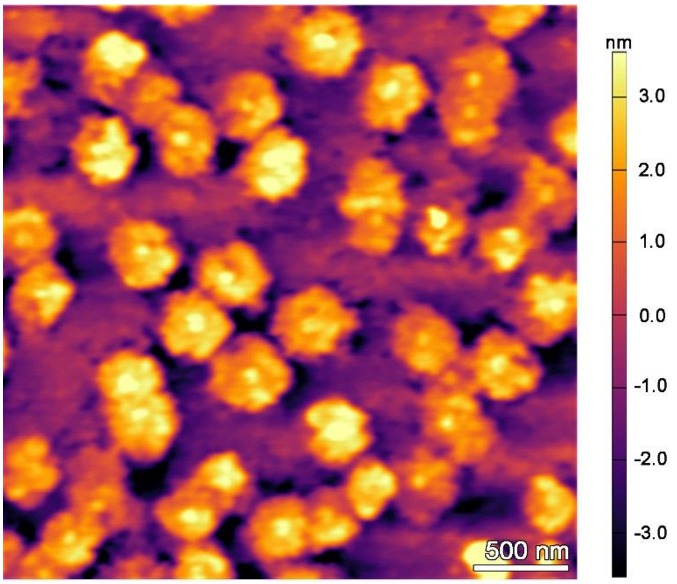

Supplementary Fig. 11| Topography image corresponding to the region in Fig. 3i-j.

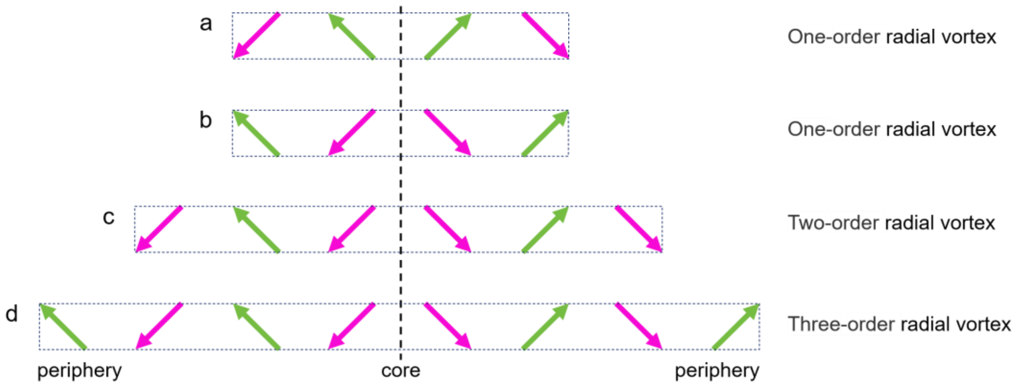

Supplementary Fig. 12| Phase difference for different multi-order radial vortices. **a,** One-order radial vortex. **b,** One-order radial vortex. **c,** Two-order radial vortex. **d,** Three-order radial vortex.

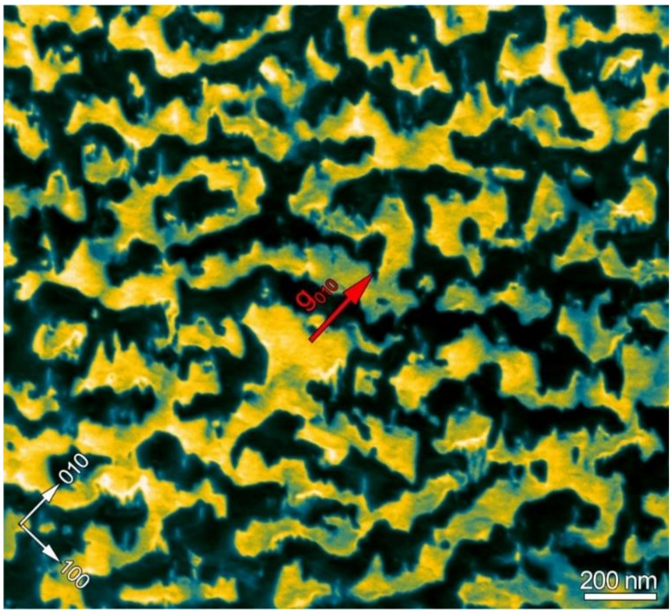

Supplementary Fig. 13| Labyrinthine domain in 20 nm BFO film.

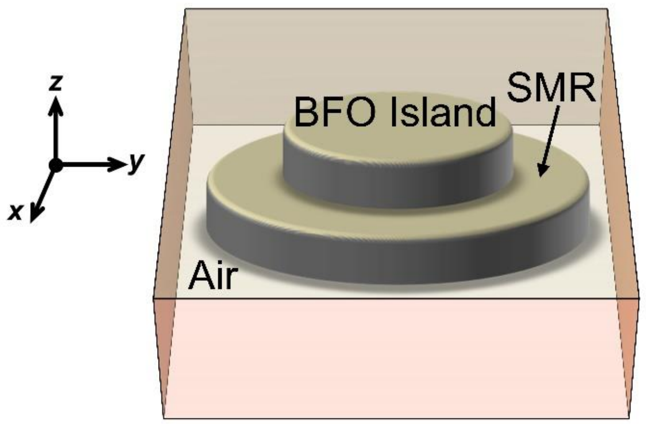

Supplementary Fig. 14| Schematic of phase-field model settings of BFO nanoisland system.

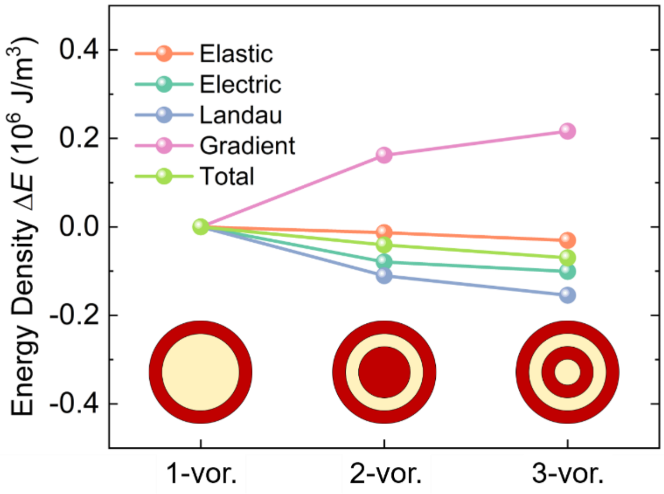

Supplementary Fig. 15| Energetics comparison of the one-order, two-order and three-order radial vortices in BFO nanostructures with diameters of 3*d* calculated from phase-field simulation. For clarity, the energy densities of the one-order radial vortex were taken as the reference. The one-order, two-order and three-order radial vortices are abbreviated to 1-vor., 2-vor. and 3-vor., respectively.

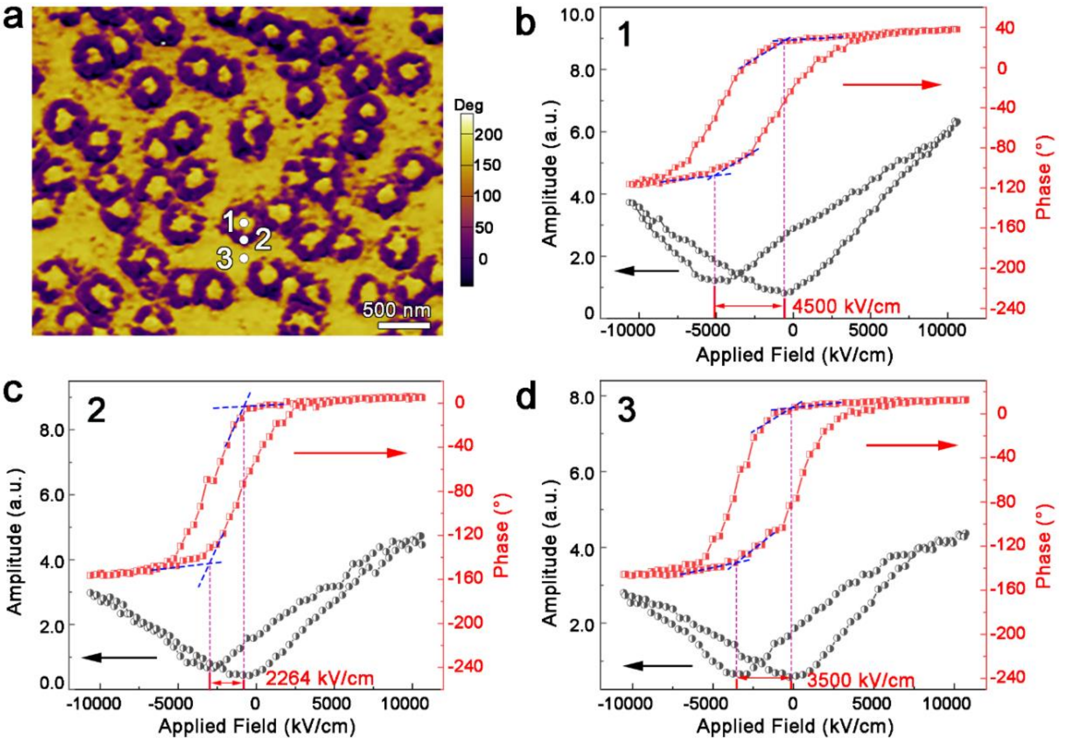

Supplementary Fig. 16| Localized polarization switching behaviors for the different regions in one two-order radial vortex. a, Vertical PFM phase image, three highlighted regions numbered as 1, 2 and 3 representing the nanoisland core, nanoisland edge and the SMR, respectively. **b-d**, Localized PFM phase-field hysteresis loops and amplitude-field butterfly loops for the three regions numbered 1, 2 and 3 in (a).

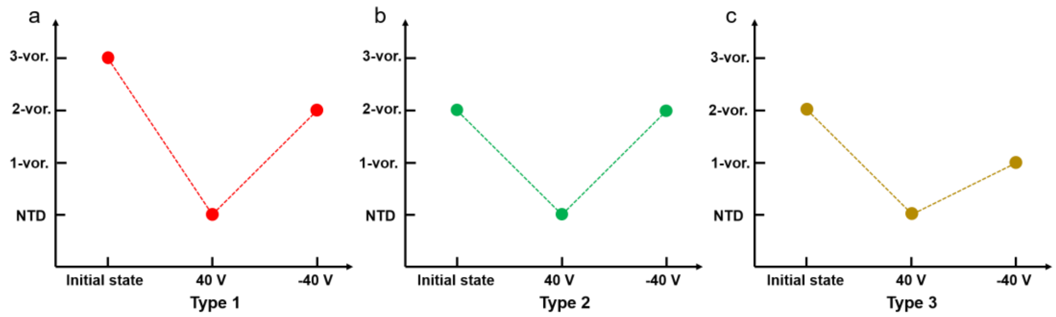

Supplementary Fig. 17| Schematic diagrams of the three kinds of topological transitions. a, Topological transition from three-order radial vortex to NTD and finally two-order radial vortex. **b,** Topological transition from two-order radial vortex to NTD and finally two-order radial vortex. **c,** Topological transition from two-order radial vortex to NTD and finally one-order radial vortex. The one-order, two-order and three-order radial vortices are abbreviated to 1-vor., 2-vor. and 3-vor., respectively.

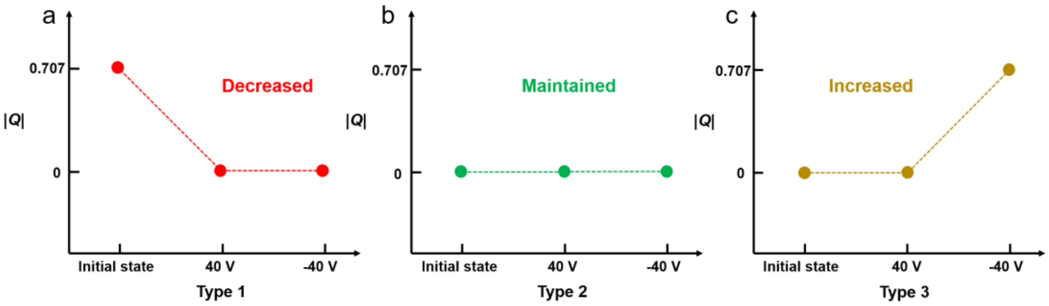

Supplementary Fig. 18| Changes of the topological charges for three kinds of topological transitions. a, Topological transition from three-order radial vortex to NTD and finally two-order radial vortex with the decreased topological charge. **b,** Topological transition from two-order radial vortex to NTD and finally two-order radial vortex with the topological charge remain unchanged. **c,** Topological transition from two-order radial vortex to NTD and finally one-order radial vortex with the increased topological charge. $|Q|$ denoting the absolute value of the topological charge.
