# Supplementary material for: Observation of multi-order polar radial vortices and their topological transition
Source: Nat Commun. 2025 Mar 21;16:2804. doi: 10.1038/s41467-025-58008-w (PMC11928551; doi:10.1038/s41467-025-58008-w)
Supplement: Supplementary file 2 — Reporting Summary [file 41467_2025_58008_MOESM2_ESM.pdf]

Corresponding author(s): Xiu-Liang Ma

Lasing Reporting Summary

Nature Research wishes to improve the reproducibility of the work that we publish. This form is intended for publication with all accepted papers reporting claims of lasing and provides structure for consistency and transparency in reporting. Some list items might not apply to an individual manuscript, but all fields must be completed for clarity.

For further information on Nature Research policies, including our data availability policy, see Authors & Referees.

Experimental design

Please check: are the following details reported in the manuscript?

1. Threshold

Plots of device output power versus pump power over a wide range of values indicating a clear threshold

☐ Yes
☒ No

Our research primarily focus on the novel ferroelectric topological domains in BiFeO3 nanostructures. The laser with fixed wavelength and energy serves only for BiFeO3 film preparation and parameters such as the threshold are not significant.

2. Linewidth narrowing

Plots of spectral power density for the emission at pump powers below, around, and above the lasing threshold, indicating a clear linewidth narrowing at threshold

☐ Yes
☒ No

Our research primarily focus on the novel ferroelectric topological domains in BiFeO3 nanostructures. The laser with fixed wavelength and energy serves only for BiFeO3 film preparation and parameters such as the linewidth narrowing are not significant.

Resolution of the spectrometer used to make spectral measurements

☐ Yes
☒ No

Our research primarily focus on the novel ferroelectric topological domains in BiFeO3 nanostructures. The laser with fixed wavelength and energy serves only for BiFeO3 film preparation and not for spectral measurements.

3. Coherent emission

Measurements of the coherence and/or polarization of the emission

☐ Yes
☒ No

Our research primarily focus on the novel ferroelectric topological domains in BiFeO3 nanostructures. The laser with fixed wavelength and energy serves only for BiFeO3 film preparation and the coherence and/or polarization of the emission are not significant.

4. Beam spatial profile

Image and/or measurement of the spatial shape and profile of the emission, showing a well-defined beam above threshold

☐ Yes
☒ No

Our research primarily focus on the novel ferroelectric topological domains in BiFeO3 nanostructures. The laser with fixed wavelength and energy serves only for BiFeO3 film preparation and parameters such as beam spatial profile are not significant.

5. Operating conditions

Description of the laser and pumping conditions
Continuous-wave, pulsed, temperature of operation

☒ Yes
☐ No

We have provided detailed descriptions of the operating conditions in the Methods section, as"pulsed laser deposition (PLD), using the Coherent ComPex PRO 201 F KrF excimer laser (λ = 248 nm)".

Threshold values provided as density values (e.g. W cm^{-2} or J cm^{-2}) taking into account the area of the device

☐ Yes
☒ No

We did not specify the threshold as a density value as this is not relevant to our study. But we have added the energy density of the laser fluence when films deposition in the Methods part, as "When growing the BFO layers, the shutter is opened, a repetition rate of 8 Hz, substrate temperature of 800 °C, oxygen partial pressure of 12 Pa and laser energy of 2 J cm-2 were used. "

6. Alternative explanations

Reasoning as to why alternative explanations have been ruled out as responsible for the emission characteristics
e.g. amplified spontaneous, directional scattering; modification of fluorescence spectrum by the cavity

☐ Yes
☒ No

Our research primarily focus on the novel ferroelectric topological domains in BiFeO3 nanostructures. The laser with fixed wavelength and energy serves only for BiFeO3 film preparation and parameters such as amplified spontaneous and directional scattering are not significant.

7. Theoretical analysis

Theoretical analysis that ensures that the experimental values measured are realistic and reasonable
e.g. laser threshold, linewidth, cavity gain-loss, efficiency

☐ Yes
☒ No

Our research primarily focus on the novel ferroelectric topological domains in BiFeO3 nanostructures. The laser with fixed wavelength and energy serves only for BiFeO3 film preparation and parameters such as laser threshold, linewidth, cavity gain-loss, efficiency are not significant.

8. Statistics

Number of devices fabricated and tested

☒ Yes
☐ No

We have fabricated four BiFeO3 films with different thicknesses to modulate the polarization distribution in the BiFeO3 nanostructures (as shown in Fig. 2, Fig. 3, Table S1, Fig. S13). Three tests were performed to confirm the polarization distribution in the nanostructures by piezoresponse force microscope, low-magnification HAADF-STEM imaging and atomic-resolved HAADF-STEM imaging. One test was performed to acquire the surface tomography by atomic force microscope.

Statistical analysis of the device performance and lifetime (time to failure)

☐ Yes
☒ No

Our research primarily focus on the novel ferroelectric topological domains in BiFeO3 nanostructures. We just deposited the BiFeO3 films as the prototype system to modulate the ferroelectric topological states. There was no statistical analysis due to no specific device in this study.
